# Dual-energy computed tomography derived pulmonary blood volume: association with pulmonary blood flow

**DOI:** 10.1186/s12931-025-03374-8

**Published:** 2025-10-22

**Authors:** Jakob Wittenstein, Rudi Apolle, Martin Scharffenberg, Carolin Rothmann, Sabine Müller, Ralf-Thorsten Hoffmann, Esther G. C. Troost, Thea Koch, Marcelo Gama de Abreu, Robert Huhle

**Affiliations:** 1 Department of Anaesthesiology and Intensive Care Medicine, Pulmonary Engineering Group, Faculty of Medicine and University Hospital Carl Gustav Carus, TUD Dresden University of Technology, Fetscherstraße 74, Dresden, 01307 Germany; 2 National Center for Tumor Diseases (NCT), Partner Site, Dresden, Germany; 3 German Cancer Research Center (DKFZ), Heidelberg, Germany; 4 Faculty of Medicine and University Hospital Carl Gustav Carus, Technische Universität Dresden, Dresden, Germany; 5 Helmholtz Association/Helmholtz-Zentrum Dresden-Rossendorf (HZDR), Dresden, Germany; 6 Institute and Policlinic for Diagnostic and Interventional Radiology, University Hospital Carl Gustav Carus Dresden at Technische Universität Dresden, Dresden, Germany; 7 Department of Radiotherapy and Radiation Oncology, Faculty of Medicine and University Hospital Carl Gustav Carus, Technische Universität Dresden, Dresden, Germany; 8 Institute of Radiooncology - OncoRay, Helmholtz-Zentrum Dresden-Rossendorf, Dresden, Germany; 9 OncoRay - National Center for Radiation Research in Oncology, Faculty of Medicine and University Hospital Carl Gustav Carus, OncoRay - National Center for Radiation Research in Oncology, Technische Universität Dresden, Helmholtz-Zentrum Dresden-Rossendorf, Dresden, Germany; 10 Partner Site Dresden, and German Cancer Research Center (DKFZ), German Cancer Consortium (DKTK), Heidelberg, Germany; 11 Department of Intensive Care and Resuscitation, Anesthesiology Institute, Cleveland Clinic, Cleveland, OH USA; 12 Department of Outcomes Research, Anesthesiology Institute, Cleveland Clinic, Cleveland, OH USA

**Keywords:** Dual-energy CT, Fluorescence labelled microspheres, Regional pulmonary perfusion, Shunt blood flow

## Abstract

**Background:**

Distribution of ventilation and pulmonary perfusion are the major determinants of pulmonary gas exchange. To study and compare strategies of mechanical ventilation in respiratory research accurate and high-resolution methods are needed to derive distribution of ventilation and perfusion with minimal additional intervention or radiation allowing repeated measurements. Dual-energy computed tomography (DECT) is an imaging technique allowing for the derivation of regional pulmonary perfused blood volume, as a surrogate for pulmonary perfusion (PP_DECT_).

Here accuracy of PP_DECT_ is evaluated in comparison to pulmonary blood flow measured with fluorescence-labeled microspheres (PP_FLM_). Its feasibility of repeated measurements is evaluated.

**Methods:**

Agreement between PP_FLM_ and PP_DECT_ was assessed by regression as well as Bland–Altman analysis in three anesthetized pigs using DECT and fluorescence labelled microspheres, respectively. Measurements were performed in two-lung and, after right sided thoracotomy, at one-lung ventilation with inhaled nitric oxide. PP_FLM_ and PP_DECT_ were assessed in three different regions of interest (ROI): the right (non-ventilated) and left (ventilated) upper and lower lung, yielding a total of 45 paired measurements over four hours. Persistent iodine accumulation was assessed by additional DECT scans before each contrast administration.

**Results:**

Regression analysis revealed a good overall association (R2 = 0.81) between PP_FLM_ and PP_DECT_, with PP_DECT_ substantially overestimating PP_FLM_ up to 30%, with limits of agreement of -18 and 18%, Low PP_FLM_ was underestimated, while high PP_FLM_ was overestimated by PP_DECT_, indicating a higher sensitivity of the later. Changes of PP_DECT_ and PP_FLM_ had a concordance of 69.4% for all measurements. Agreement and concordance were highest in ventilated and lowest in non-ventilated ROIs. No persistent iodine enhancement was detected in the lung parenchyma after repetitive measurements per hour.

**Conclusions:**

Dual-energy CT based measurement of pulmonary perfusion shows promising results indicating its feasibility in translational research on strategies of mechanical ventilation.

## Introduction

Distribution of ventilation and pulmonary perfusion are the major determinants of pulmonary gas exchange. Especially in the diseased lung ventilation-perfusion matching can be deteriorated resulting in poor gas exchange and may contribute to so-called ventilator-induced lung injury (VILI). In fact, it has been claimed that the interruption of pulmonary blood flow, combined with ongoing mechanical ventilation is a mechanism of VILI [13]. Therefore, information on regional distribution of pulmonary perfusion and ventilation offers valuable physiological insights into acute lung pathophysiology, and has an important role in clinical and experimental research [6].

In experimental research, where repeated measurements of pulmonary perfusion and ventilation are needed, fluorescence-labelled microspheres (FLM) are used as a gold standard to measure pulmonary perfusion [10]. However, this method features low resolution, high degrees of additional errors due to manual analysis, and requires labor-intensive tissue processing including postmortem dissection of the lung, limiting its applicability to animal models.

Dual-Energy Computed Tomography (DECT) is an imaging technique for producing quantitative iodine density images and depicting the distribution of regional pulmonary perfused blood volume, and may be regarded as a surrogate for pulmonary perfusion. It is widely used in the clinical setting as a surrogate for pulmonary perfusion measurement [1, 4, 11, 14, 24].

In the present study, we compare the distribution of perfused pulmonary blood volume measured by DECT (PP_DECT_) and pulmonary blood flow measured by FLM (PP_FLM_), respectively. Both are herein considered as surrogates for pulmonary perfusion and are assessed during different conditions of ventilation and perfusion in pigs and describe a method to derive ventilation-perfusion maps from DECT and static CT imaging.

In this analysis we use data of a yet unpublished experimental study in pigs on the impact of inhaled nitric oxide on gas exchange during one-lung ventilation.

We hypothesized that DECT based assessment of pulmonary blood volume combined with tidal volume change images derived from co-registered static CT scans is able to measure and track changes of pulmonary perfusion.

## Materials and methods

This study was conducted as a secondary investigation of another experimental study in three female pigs (German landrace, weighing 33.7 to 42.5 kg). The Institutional Animal Care and Welfare Committee and the Government of the State of Saxony, Germany, approved the main and secondary protocols (25–5131/496/33). All animals received humane care in compliance with the Principles of Laboratory Animal Care formulated by the National Society for Medical Research and the US National Academy of Sciences Guide for the Care and Use of Laboratory Animals.

### Experimental protocol

The data analysed is derived as defined in the protocol of the main study on the effects of inhaled nitric oxide on gas exchange during one-lung ventilation during right-sided pneumothorax to model thoracic surgery. In addition, for this secondary investigation fluorescence labelled microspheres were administered to derive pulmonary blood flow at each time point (as described below).

**Fig. 1:**
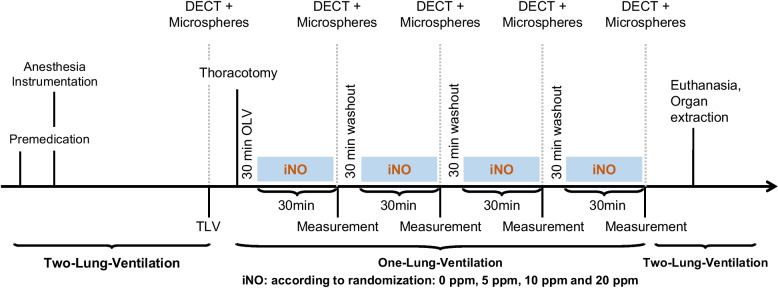
Time course of measurements and interventions. iNO: inhaled nitric oxide, DECT: dual-energy computed tomography

The time course of experiments and interventions of the main study is shown in Fig. 1. Intravenous anesthesia and muscle paralysis were induced and maintained with midazolam. Lungs were ventilated with a fraction of inspired oxygen of 1.0, tidal volume (VT) of 7 ml kg^−1^ during two-lung ventilation and of 5 ml kg^−1^ during one-lung ventilation (OLV), positive end-expiratory pressure (PEEP) of 5 cmH_2_0, and respiratory rate (RR) adjusted to achieve arterial pH > 7.25 allowing permissive hypercapnia.

For hemodynamic monitoring and blood gas analysis, an 8.5 Fr. sheath (Arrow International, Reading, PA, USA) was inserted in the right internal carotid artery and a 7.5 Fr. pulmonary artery catheter (Opticath, Abbott, Abbott Park, USA) was advanced through an 8.5 Fr. sheath, placed in the right external jugular vein. Thoracic surgery was mimicked as previously described [8].

Animals were randomized to one of four sequences of different concentrations of inhaled nitric oxide (iNO) during OLV: iNO = 0 ppm, iNO = 5 ppm, iNO = 10 ppm and iNO = 20 ppm.

### Imaging

In summary, the imaging protocol described here consisted of CT scans at end-expiration and end-inspiration to determine distribution of ventilation. Distribution of pulmonary blood volume was assessed from one DECT scan at mean-lung volume.

#### Protocol

**Fig. 2:**
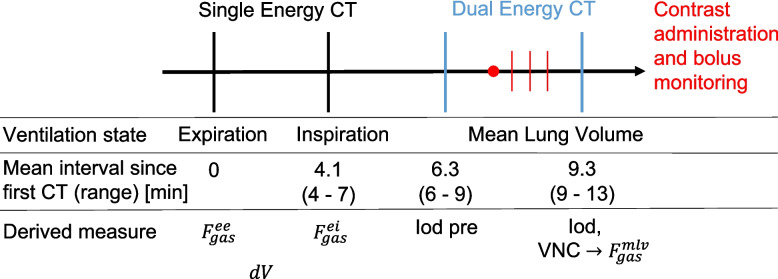
Imaging protocol of Dual-Energy Computed Tomography (DE)CT as performed at mean-lung volume for each measurement time point, its timing and derived measures with: gas fractions at end-expiration, end-inspiration and mean-lung volume (${F}_{gas}^{ee},{F}_{gas}^{ei}$ and ${F}_{gas}^{mlv}$), respectively; tidal gas volume difference (dV); virtual non-contrast (VNC), DECT for tracer enrichment analysis (Iodine pre); and DECT following tracer injection

Following induction of anesthesia and instrumentation, animals were subjected to the imaging protocol (Fig. 2) at two-lung ventilation as well as 30 min after each start of administration of respective concentration of inhaled nitric oxide (iNO) of 0, 5, 10 and 20 ppm during left-sided one-lung ventilation (OLV) in each animal.

#### Perfused pulmonary blood volume

DECT was acquired with a SOMATOM Definition Edge scanner (Siemens Healthineers, Erlangen, Germany) and performed using a split filter (gold/tin) introduced into the beam to deliver different X-ray spectra to different parts of the detector (Twin Beam feature). Spiral scans were acquired at a tube potential of 120kVp and with current modulation set to a reference current time product of 384mAs. Axial fields of view typically measured around 330 mm and were scanned over approximately 9 s (pitch factor: 0.35). Images were reconstructed into 1mm^3^ isotropic voxels using the Qv40f kernel. Iodine contrast agent (Ultravist 370; Bayer Healthcare, Leverkusen, Germany, 60 ml, flow rate of 4 ml/s) was injected prior to DECT acquisition using a MEDRAD Stellant injector (Bayer Healthcare, Leverkusen, Germany). Thereafter, a 50 ml saline flush at the same rate was performed. From the start of injection a thick monitoring slice centered on the right atrium was acquired every second until contrast agent could be identified by the operator. Scan acquisition was then started after an additional delay of 5 s. DECT was acquired at mean lung volume.

Iodine enhancement images directly related to relative perfused pulmonary blood volume were derived as a surrogate for pulmonary perfusion (PP_DECT_). Iodine images were derived from DECT images using a proprietary algorithm (Virtual Unenhanced, syngo.via, version VB40B_HF01; Siemens Healthineers). The algorithm performs a post-reconstruction material decomposition into water (virtual non-contrast—VNC) and iodine images [7, 22], where each is supposed to not contain the signal of the other. The underlying assumption of this algorithm is that VNC and iodine images are derivable from low- and high energy images, such that their sum equals a mixed image with minimal noise. Derived iodine images then, in particular, are meant to only contain information about the additional X-ray attenuation afforded by the iodine contrast agent beyond that caused by the unenhanced tissue.

More radio-dense structures, such as cortical bone, will generally also be present in the iodine images, but their signal will lie outside the regions of interest evaluated for PP_DECT_ determination. Iodine images yield voxel values in terms of attenuation (HU) which is proportional to the iodine concentration. The algorithm’s sole configurable parameter, the iodine ratio, relates the X-ray attenuation effected by iodine in the low-energy to that in the high-energy spectrum. It is independent of the iodine concentration and was set to 1.46 for all decompositions.

Segmentation of the lung was performed as described above based on the native reconstruction at mean lung volume. Pulmonary vessels were segmented and extracted by local threshold region-growing algorithm in the DECT image using 3D Slicer [8]. This enabled the construction of parenchymal regions of interest which excluded large vessels and airways. Assuming a constant iodine concentration throughout the scanned blood volume the iodine images give a signal, which is proportional to the fractional blood content of each voxel. Normalisation with respect to the blood iodine concentration from a suitable reference region (e.g. a large vessel) can be done and integration over the lung parenchyma would yield the perfused pulmonary blood volume. In this study, however, iodine images were normalised by the sum over the entire lung parenchyma, such that the sum over a particular region of interest gave its fraction of the overall perfused blood volume. While conceptually different, perfused blood volume was investigated as a surrogate for pulmonary blood flow, or pulmonary perfusion in general, and hence termed PP_DECT_ throughout the remainder of this work.

With only one hour between contrast agent administrations, there is a risk of persistent iodine enhancement through tracer accumulation in the lung parenchyma. This was investigated through an additional DECT scan performed just prior to contrast administration (Fig. 2), upon which the same iodine quantification (Iod pre) was performed, left and right lung mean HU in VNC as well as in Iod images during OLV were calculated and compared.

#### Distribution of ventilation

Static whole lung spiral computed tomography (SOMATOM Definition Edge scanner, Siemens Healthineers AG, Erlangen, Germany; tube potential 120 kVp, reference exposure for current modulation 250 mAs, reconstruction kernel Br58f, voxel size 1 × 1×1 mm^3^) was acquired during end-inspiratory and end-expiratory hold of approx. 10 s at end-inspiratory and end-expiratory airway pressure. Gas fractions were derived from CT values in Hounsfield units (HU) by ${F}_{gas}=\frac{HU}{-1000}$. Scanner quality assurance (QA) was carried out monthly according to manufacturer recommendations and local regulatory requirements. The CT number of water was assessed monthly with an accuracy with-in 4 HU and the CT number of air was re-adjusted daily during routine gain calibration. CT number linearity with-in the range of interest was performed 9 months before the experiments using two surrogate materials as described in [17].

Lung segmentation in a semi-automatic approach: 1 – automatic segmentation of the end-inspiratory scan lung parenchyma by a deep convolutional neural network [12]; 2 – manual refinement to include atelectatic lung regions (ITKSnap, www.itksnap.org, [28]; 3 – segmentation and exclusion of big airways up to the fivfifthed generation by region-growing and local threshold (3Dslicer, www.slicer.org, [8]; 4 – co-registration (see details below of the resulting mask to end-expiratory volume; and final manual refinement.

Images of tidal volume change ($dV$) were determined following co-registration of the static end-inspiratory gas fractions (${F}_{gas}^{ei}$) to the gas fraction at end-expiratory lung volume (${F}_{gas}^{ee}$) using the Advanced Normalization Toolkits ANTs [3]. The end-inspiratory lung mask was smoothed and expanded by morphologically opening (radius of 2 voxel), closing (15 voxel), dilation (10 voxel). The resulting mask was applied to Gaussian de-noised ${F}_{gas}^{ei}$ image (3 × 3x3 voxel Gaussian kernel). Image registration was performed using diffeomorphic transforms with BSpline (distance 26 voxels) spatial multistage regularization (100 × 100x70 × 50x0 iterations per stage, 10^–6^ convergence threshold, 10 iterations convergence window, shrink factors 10 × 6x4 × 2x1, smoothing sigmas 5 × 3x2 × 1x0 voxel) and ANTs neighborhood cross correlation as convergence metric (radius 4 voxel). $\dot{V}$ was then determined at end-expiratory lung volume by $dV= {F}_{gas,ei}^{@ee}\left|J\right|-{F}_{gas}^{ee}$, with the Jacobian determinant |J| and end-inspiratory gas fraction co-registered to end-expiratory gas fraction denoted by ${F}_{gas,ei}^{@ee}$.

### Pulmonary blood flow

In summary, pulmonary blood flow is derived as a ground-truth for pulmonary perfusion from Fluorescence Labelled Microspheres. Since direct voxel-based alignment of PP_FLM_ and PP_DECT_ was not possible due to the limitation of the FLM method, the comparison is done based on three regions of interest.

Distribution of regional pulmonary blood flow, also termed pulmonary perfusion (PP_FLM_) was determined using IV-administered fluorescence and color-labelled microspheres, 15 µm in diameter [15]. The colors used were blue (excitation/emission wavelength: 365/415 nm), blue-green (430/465 nm), yellow-green (480/507 nm), scarlet (660/680 nm), and red (570/590 nm) [21]. To avoid bias, the colors were randomly assigned at any given time point. Immediately before injection, the microspheres were vortexed, sonicated for 90 s, and drawn into 2 ml syringes. All the injections were performed for over 60 s to average the blood flow over several cardiac and respiratory cycles. During the injection, ∼1.5 × 106 microspheres were administered. Postmortem lungs were extracted en bloc and flushed with 50 ml kg^−1^ of a hydroxyethyl starch 130/0.4 solution (Voluven, Fresenius Kabi, Bad Homburg, Germany) and air-dried by continuous tracheal airflow for seven days with a continuous pressure of 25 cm H_2_O. The lungs were then coated with one-component polyurethane foam (4W VARIO Foam, BTI Befestigungstechnik GmbH & Co. KG, Ingelfingen, Germany), suspended vertically in a square box, and embedded in rapidly setting PU-System 2-component polyurethane foam (polyetherpolyol, polypropylenglycole and diphenylmethane diisocyanate, isomers, homologues isocyanate; VOSSCHEMIE GmbH, Germany). The foam block was then cut into cubes of 12 × 12x12 mm. Each cube belonging to the lung, after exclusion of large airways, was weighed and assigned a three-dimensional coordinate. The samples were then soaked for two days in 3 ml of 2-ethoxyethyl acetate (Aldrich Chemical Co. LLC, Milwaukee, WI, United States) to retrieve the fluorescent dye. The fluorescence was read in a SYNERGY Mx microplate reader (Agilent BioTek, Charlotte, Vermont, USA). The measured intensity of fluorescence in each probe (Imager Software Gen5 Version 3.12, Agilent BioTek, Charlotte, Vermont, USA) was normalized according to its weight (x_i_).

### Statistical analysis

Data are presented as mean ± standard deviation or median [1st…3rd quartile].. No tests for normal distribution have been performed due to the limited sample size.

Agreement between both measurement techniques was assessed by pulmonary perfusion in three ROIs per scan: right lung, left caudal and left cranial regions. Right and left lung were manually delineated. The left lung ROI was separated in axial direction such that both left lung ROIs were equally sized (equal volume). PP_DECT_ was divided by tissue mass derived from the respective VNC image. Both, PP_DECT_ and PP_FLM_ were normalization by its sum per measurement/scan.

Association between both methods was analyzed by regression (slope S and intersect) and Bland–Altman analysis, bias and limits of agreement (LoA).

Concordance analysis was performed with the aim to elucidate, how well the change of pulmonary perfusion from one measurement to the next is covered by the two methods compare. To this end, changes of PP_FLM_ and PP_DECT_ were calculated, their association assessed by regression analysis, and visualized. Concordance was quantified by dividing the number of changes of PP of both methods increased or decreased (quadrants I and III) by the number of PP changes where measurements by one method increased/decreased and the other decreased/increased, respectively (quadrants II and IV).

Iodine enrichment mean ROI values for native and contrast enhanced scans were tested using repeated measures ANOVA applied to a linear-mixed effects model with factors scan (native or enhanced), aeration state (aerated vs. non-aerated) and acquisition time (hours) since first scan during two-lung ventilation (NLME package) [18, 19]. Statistical significance was accepted at *P* < 0.05. Analyses were conducted with R statistical programming language [20]. Graphs were prepared using Matlab (Mathworks Inc, CA, USA) and R.

## Results

Gas exchange, hemodynamics, and respiratory variables are depicted in Table 1.

**Table 1:** Gas exchange, haemodynamics and respiratory variables for the five interventions two lung ventilation (TLV) and one-lung ventilation with different concentrations of inhaled NO

	TLV	iNO_0_	iNO_5_	iNO_10_	iNO_20_
pHa	7.44 ± 0.02	7.34 ± 0.01	7.30 ± 0.03	7.35 ± 0.01	7.32 ± 0.04
PaCO_2_ [mmHg]	53 ± 5	65 ± 3	72 ± 1	66 ± 5	70 ± 5
PaO_2_ [mmHg]	467 ± 29	123 ± 29	177 ± 120	281 ± 107	112 ± 10
MAP [mmHg]	79 ± 15	85 ± 22	82 ± 24	97 ± 14	84 ± 18
HR [min^−1^]	108 ± 34	129 ± 28	135 ± 33	115 ± 12	153 ± 36
CO [l min^−1^]	5.0 ± 0.3	4.2 ± 0.9	5.7 ± 3.2	4.2 ± 1.3	6.0 ± 0.9
RR [min^−1^]	23 ± 3	31 ± 1	31 ± 1	31 ± 1	31 ± 1
V_T_ [ml kg^−1^]	7.7 ± 0	5.4 ± 0.1	5.4 ± 0.1	5.4 ± 0.1	5.4 ± 0.1
PEEP [cmH_2_O]	5 ± 0	5 ± 0	5 ± 0	5 ± 0	5 ± 0

Data as mean ± standard deviation; with: 0 ppm (iNO_0_), 5 ppm (iNO_5_), 10 ppm (iNO_10_), and 20 ppm (iNO_20_). pHa, arterial pH; PaCO_2_, arterial partial pressure of carbon dioxide; PaO_2_, arterial partial pressure of oxygen; MAP, mean arterial pressure; HR, heart rate; CO, cardiac output; RR, respiratory rate; VT, tidal volume; PEEP, positive end-expiratory pressure

### Pulmonary perfusion

**Fig. 3:**
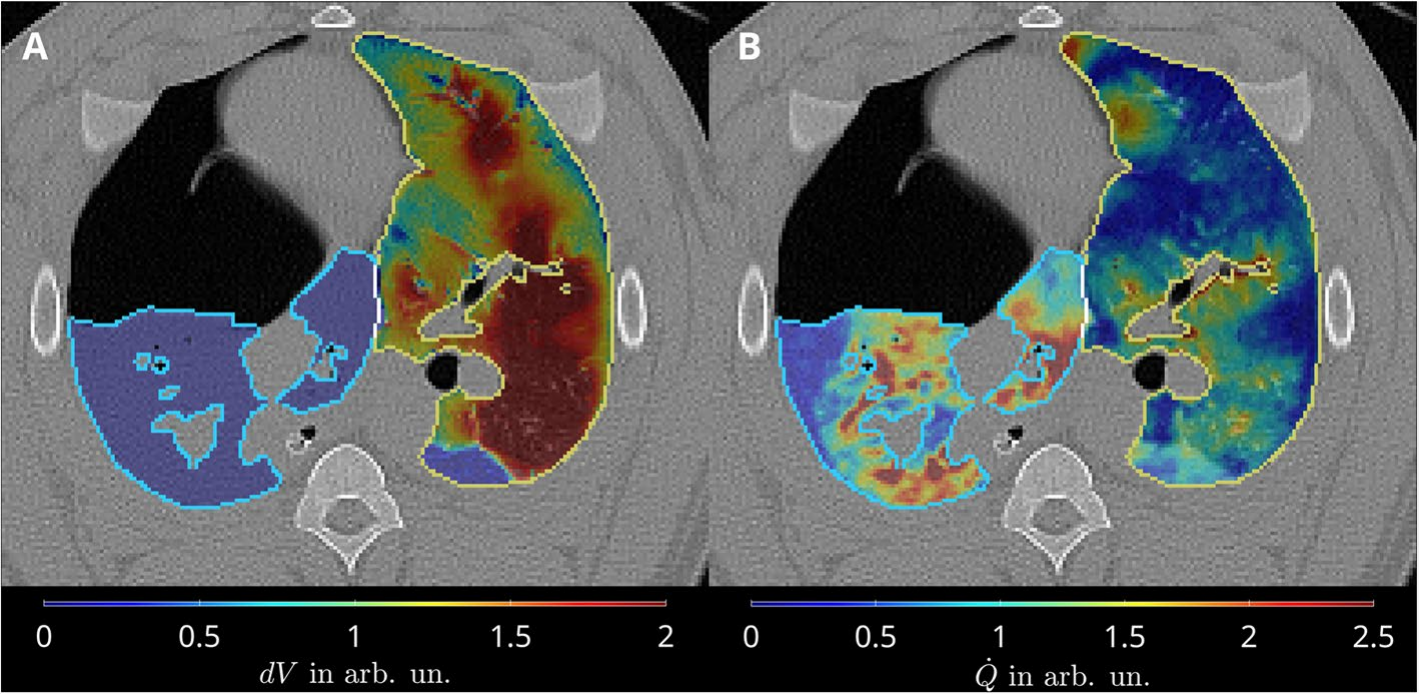
Representative transversal slides during one-lung ventilation in the left lung and its absence in the right lung (**A**) and pulmonary perfusion from DECT ${PP}_{DECT}$ in both lungs (**B**)

**Fig. 4:**
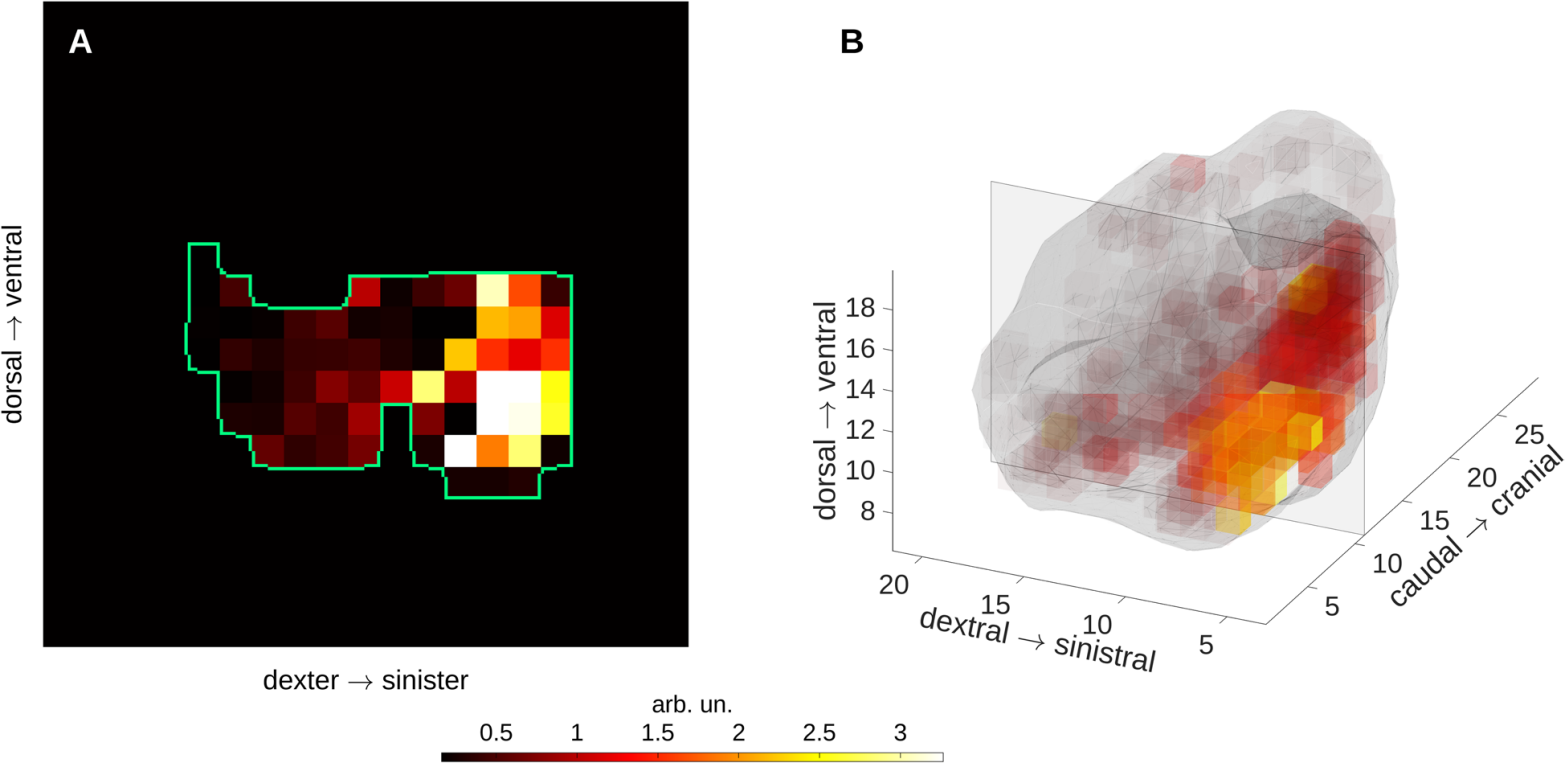
Representative distribution of PP_FLM_ shows the low resolution and substantial deviation from lung shapes due to the fluorescence labelled-microspheres analysis; central transversal slice between most caudal and most cranial lung (left) and a three-dimensional representation of PP_FLM_ for the same scan seen from dorsal

A representative image of relative pulmonary blood volume is shown in Fig. 3B. A representative FLM-based PP_FLM_ image of the same experiment/timepoint can be found in Fig. 4.

**Fig. 5:**
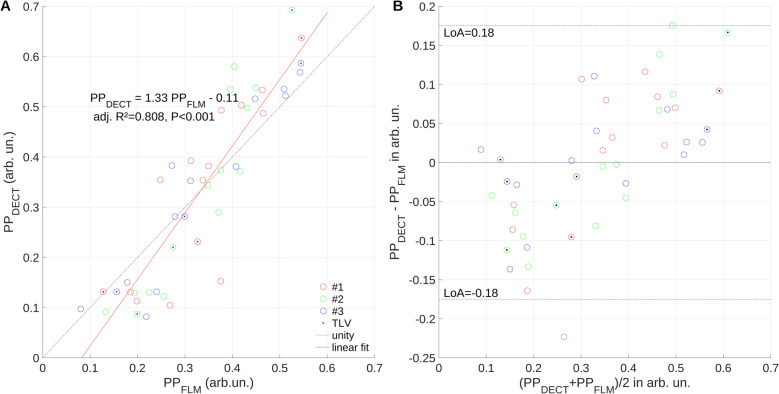
Regression analysis (**A**) and Bland–Altman plot (**B**) suggest systematic under-/over-estimation of low/high pulmonary blood flow (PP_FLM_) by perfused pulmonary blood volume (PP_DECT_); limits of agreement (LoA), number of experiment (#)

**Fig. 6:**
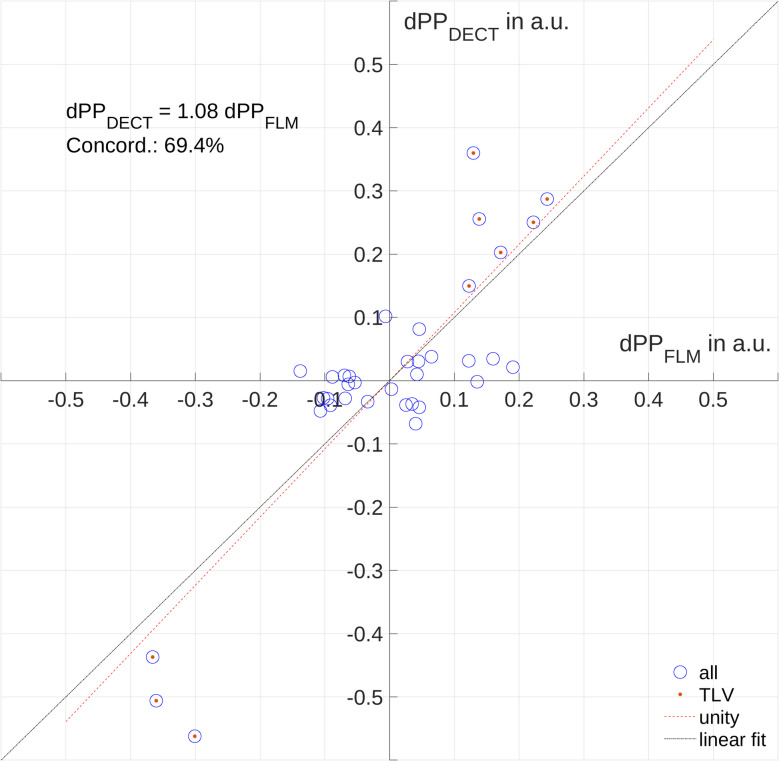
Concordance plot of perfused pulmonary blood volume (PP_DECT_) determined by DECT imaging and pulmonary blood flow (PP_FLM_) as derived by Fluorescence-labelled microspheres. Change of PP_DECT_ and PP_FLM_ between measurements (dPP_DECT_ and dPP_FLM_, respectively), TLV, two lung ventilation

Overall experiments, measurements and ROIs, regression analysis (Fig. 5A) revealed a good association (coefficient of determination adj. R2 = 0.81) between PP_FLM_ and PP_DECT_, however the later overestimating the former by 33% with limits of agreement of −18 and 18% (Fig. 5B). Changes of pulmonary blood volume and flow, respectively, had a concordance of 69.4% for all measurements (Fig. 6), indicating that in approximately 70% of measurements the same direction of change was captured by both methods.

R2 and concordance were higher for two-compared to one-lung ventilation (0.92 vs. 0.75 and 100 vs. 59%, Table 2). In the right lung, independently of ventilation, S, R2 and absolute bias were highest (S = 1.32, R2 = 0.816, Bias 0.06) while concordance was lowest (66%). Conversely, in the left cranial ROI, R2 = 0.611, Bias = 0.01 and LoA = 0.13 were lowest while concordance (75%) was highest and slope was closest to unity (S = 0.95). When considering both left-lung ROIs (cranial and caudal ROI) together during one and two lung ventilation, slope was 1.18.

**Table 2:** Parameters of regression and concordance analysis of pulmonary blood volume and pulmonary blood flow for all regions of interest and measurement points (3 per scan, 3 × during two-lung and 12 × during one-lung ventilation)

Ventilated Lung	ROI	Slope S	Intersect	adj. R^2^	P	Bias	LoA	Concordance
TLV	all	1.38	−0.12	0.919	< 0.001	0	0.175	100
OLV		1.30	−0.10	0.754	< 0.001	0	0.178	59
all	cranial	0.95	0.21	0.611	< 0.001	0.01	0.129	75
	caudal	1.27	0.07	0.648	< 0.001	0.05	0.189	66
	right	1.32	−0.14	0.816	< 0.001	−0.06	0.145	66
all	all	1.33	−0.11	0.808	< 0.001	0	0.175	69

One-lung ventilation (OLV), Two lung ventilation (TLV), Region of interest (ROI), and Limits of agreement (LoA); all values in arbitrary units after normalization to the sum

### Iodine accumulation analysis

**Fig. 7:**
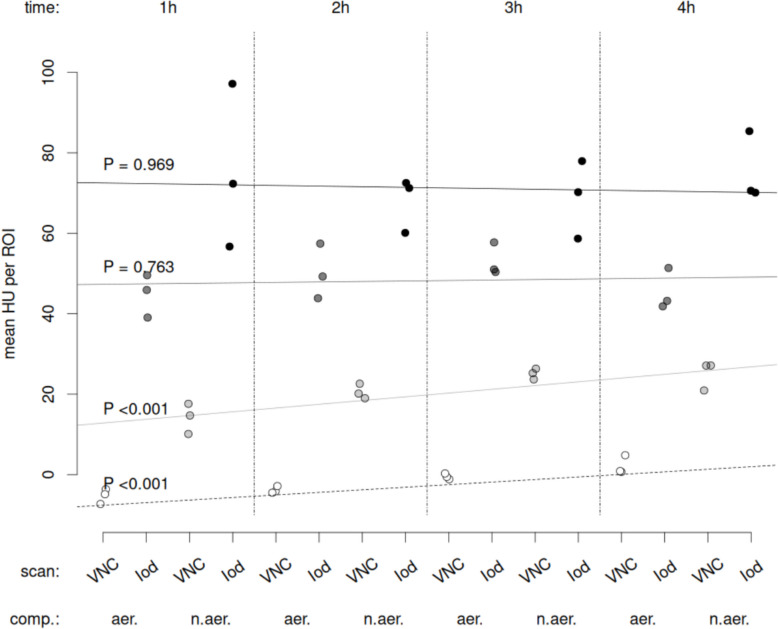
Mean Hounsfield unit (HU) for VNC native images (light grey and white circles) as well as respective Iodine images (dark grey and black circles) in aerated (aer.) left and non-aerated (n.aer.) right lung (comp.), respectively; VNC: virtual non-contrast image

Mean normalized lung iodine enhancement (HU) in native images increased with time both for aerated and non-aerated lung regions (*P* < 0.001, each). However, no such time dependence was evident for enhanced images independently from the aeration state (Fig. 7).

### Distribution of pulmonary perfusion and tidal volume change

**Fig. 8:**
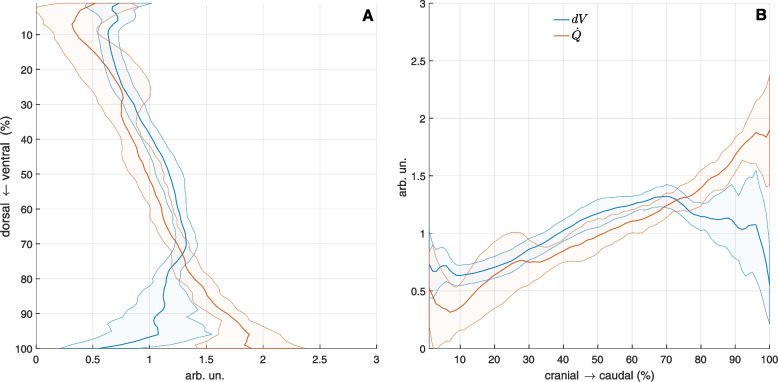
Ventilation (dV) is higher than perfusion $\dot{Q}={PP}_{DECT}$ in gravity independent lung regions (ventral, cranial) and lower in dependent lung regions (dorsal, caudal), respectively

Representative transversal slides of pulmonary perfusion and tidal volume change *dV* during one-lung ventilation is depicted in Fig. 3. Distributions of $dV$ and ${\dot{Q}=PP}_{DECT}$ along ventro-dorsal and caudal-cranial axes are shown in Fig. 8. While ventral and cranial regions showed high relative volume changes and low relative perfusion resulting in functional dead space, dorsal and caudal regions were characterized by low relative volume changes and high relative perfusion resampling intra-pulmonary shunt. Equal relative volume change and perfusion was localized in central lung regions.

## Discussion

The main findings of this study were that pulmonary perfusion measured by DECT, compared with FLM: 1) was within limits of agreement of −18 and 18%; 2) underestimated low pulmonary perfusion and overestimated high pulmonary perfusion 3) overestimated lung regions with high changes in aeration and had the best agreement in lung regions with low aeration changes; and 4) tracked the same direction of change in pulmonary perfusion in 69.4% of all measurements.

In this study we investigated DECT ability to measure and track changes of the distribution of pulmonary perfusion of the whole lung compared to intravenous microspheres, chosen as reference, a method well-described yet with considerable limitations, which allows repeated imaging of the entire lung with high spatial resolution [10]. The main weaknesses of microspheres based pulmonary perfusion measurement are the low spatial resolution and the labor-intensive and thus error prone analysis, increasing stochastic error and limited preservation of anatomic shapes and features.

Furthermore, the used animal model of two-lung and one-lung ventilation with different concentrations of iNO resembled distinct yet extreme conditions of ventilation/perfusion matching and thereby covered clinically important extremes of gas exchange impairment usually seen in patients with acute lung failure. Additionally, we used the gold standard method to determine cardiac output, namely transpulmonary thermodilution using a pulmonary artery catheter, ensuring that cardiac output was constant at the respective DECT and FLM measurements.

Our finding that pulmonary perfusion measured by DECT, compared with FLM, showed a good overall association and was within limits of agreement of −18 and 18% is in line with experimental [5, 9, 26, 29, 30] and clinical studies [16, 23, 27] using dynamic multi-detector CT and SPECT-CT as a comparator. However, these investigations came with limitations: While SPECT measurements have limited spatial resolution, multi-detector CT allows to investigate only a small field of view precluding determination of perfusion of the whole lung, which limits generalizability of these results.

We observed that DECT, compared to FLM, overestimated pulmonary perfusion in the right lung, where arguably the highest aeration changes occurred when switching from two-lung (aerated and ventilated) to one-lung ventilation (non-aerated and non-ventilated). Conversely, highest agreement was observed in the cranial left lung that was subject to the lowest aeration changes within the study as an aerated and ventilated non-dependent lung region. A simultaneous increase in lung region density and pulmonary perfusion may have detrimental effects on the performance of the applied material decomposition algorithm and thus lead to an overestimation of true changes in pulmonary perfusion using DECT.

Despite the systematic differences, DECT detected the direction of change in pulmonary perfusion. Amongst the different conditions DECT performed best when changing from two-lung to one-lung ventilation, while concordance was lower during one-lung ventilation with different concentrations of iNO. This is not unexpected as pulmonary perfusion is substantially affected by the switch from two-lung to one-lung ventilation, while iNO has only minor effects on distribution of perfusion [25].

A variety of DECT implementations are now available, ranging in sophistication from the sequential acquisition of two scans (dual spiral DECT) to systems employing two separate tube-detector pairs on the same gantry (dual source DECT). Their performance for iodine quantification is primarily determined by their energy separation. The more distinct the two spectra can be made to be, the more effectively can materials be differentiated. This is reflected in the iodine ratio, which had a rather modest value of 1.46 for the Twin Beam scanner employed in this study, whereas it reaches values twice as high with techniques employing two separate tube potentials [2]. Temporal coherence, require that regions sampled by one spectrum are still in the same place when sampled by the second for meaningful dual energy calculations to be performed. This is most easily accomplished with dual source machines, but Twin Beam scanners and those which rapidly switch between low and high tube potentials achieve near instantaneous acquisitions with both spectra, as well. The problem also does not present when scanning stationary objects, a condition which was mostly achieved in this study by acquiring DECT during controlled breath holds. Slightly mismatched phases of the cardiac cycle seen in the two spectra result in some residual misalignment, however, which might explain the sometimes spurious iodine signals seen at lung-heart interfaces. Overall, dual source scanners might be best placed to conduct DECT-based perfusion measurements with iodine contrast in patients. A Twin Beam method might be preferable despite its modest spectral separation due to limitations in the transverse field of view of dual source scanners.

Our study knows several limitations. First, the study protocol and interventions (NO inhalation and pneumothorax) that led to the alterations in pulmonary perfusion analyzed here, were selected and defined in another experimental study and as such may not be ideal for the investigation at hand. Second, the spectral separation for DECT was low, compared to other studies, which might affect the results presented here. Third, the choice of DECT implementation [2] as well as the temporal coherence of DECT might have affected our results. Fourth, the intervention during one-lung ventilation measurements, namely the different concentrations of iNO, might have induced a change in pulmonary perfusion being within the stochastic error of both methods. Fifth, the FLM technique precluded the manual, and somehow arbitrary definition of ROIs since ventilation status cannot be exactly reproduced in a postmortem measurement. Sixth, animals were mechanically ventilated with a fraction of inspired oxygen of 1.0 throughout the experiment, potentially promoting resorption atelectasis.

## Conclusion

In conclusion, the agreement between DECT and FLM for measuring and tracking changes of pulmonary perfusion was moderate, with DECT being more sensitive and with lower scatter. The absence of persistent iodine enhancement in the lung parenchyma indicates the feasibility of repeated perfusion measurements.

## Data Availability

Data and algorithm will be made available upon request to the corresponding author.

## Abbreviations

DECT: Dual-energy computed tomography
FLM: Fluorescence-labelled microspheres
HU: Hounsfield units
iNO: Inhaled nitric-oxide
LoA: Limits of agreement
OLV: One-lung ventilation
PP_DECT_: Perfused pulmonary blood volume determined by DECT
PP_FLM_: Pulmonary blood flow measured using FLM
Q: Perfusion
ROI: Region of interest
TLV: Two-lung ventilation
VNC: Virtual non-contrast
